# Association Between Screen Time Exposure in Children at 1 Year of Age and Autism Spectrum Disorder at 3 Years of Age: The Japan Environment and Children’s Study

**DOI:** 10.1001/jamapediatrics.2021.5778

**Published:** 2022-01-31

**Authors:** Megumi Kushima, Reiji Kojima, Ryoji Shinohara, Sayaka Horiuchi, Sanae Otawa, Tadao Ooka, Yuka Akiyama, Kunio Miyake, Hiroshi Yokomichi, Zentaro Yamagata, and the Japan Environment and Children’s Study Group

**Affiliations:** 1 Center for Birth Cohort Studies, University of Yamanashi, Chuo, Yamanashi, Japan; 2 Department of Health Sciences, University of Yamanashi, Chuo, Yamanashi, Japan

## Abstract

**Importance:**

It is unclear to what extent the duration of screen time in infancy is associated with the subsequent diagnosis of autism spectrum disorder.

**Objective:**

To examine the association between screen time in infancy and the development of autism spectrum disorder at 3 years of age.

**Design, Setting, and Participants:**

This cohort study analyzed data from mother-child dyads in a large birth cohort in Japan. This study included children born to women recruited between January 2011 and March 2014, and data were analyzed in December 2020. The study was conducted by the Japan Environment and Children’s Study Group in collaboration with 15 regional centers across Japan.

**Exposures:**

Screen time at 1 year of age.

**Main Outcomes and Measures:**

The outcome variable, children diagnosed with autism spectrum disorder at 3 years of age, was assessed using a questionnaire administered to mothers of the participating children.

**Results:**

A total of 84 030 mother-child dyads were analyzed. The prevalence of children with autism spectrum disorder at 3 years of age was 392 per 100 000 (0.4%), and boys were 3 times more likely to have been diagnosed with autism spectrum disorder than were girls. Logistic regression analysis showed that among boys, when “no screen” was the reference, the adjusted odds ratios were as follows: less than 1 hour, odds ratio, 1.38 (95 % CI, 0.71-2.69; *P* = .35), 1 hour to less than 2 hours, odds ratio, 2.16 (95 % CI, 1.13-4.14; *P* = .02), 2 hours to less than 4 hours, odds ratio, 3.48 (95% CI, 1.83-6.65; *P* < .001), and more than 4 hours, odds ratio, 3.02 (95% CI, 1.44-6.34; *P* = .04). Among girls, however, there was no association between autism spectrum disorder and screen time.

**Conclusions and Relevance:**

Among boys, longer screen time at 1 year of age was significantly associated with autism spectrum disorder at 3 years of age. With the rapid increase in device usage, it is necessary to review the health effects of screen time on infants and to control excessive screen time.

## Key Points

**Question:**

Is screen-time duration in children at 1 year of age associated with autism spectrum disorder at 3 years of age?

**Findings:**

A total of 84 030 mother-child dyads were analyzed using data derived from a large birth cohort study conducted in Japan. Among boys, but not girls, longer screen time at 1 year of age was significantly associated with autism spectrum disorder diagnosis at 3 years of age.

**Meaning:**

Guidance on appropriate screen time in infancy is recommended.

## Teaser

This cohort study examines the association between screen time in infancy and the development of autism spectrum disorder at 3 years of age.

## Introduction

Autism spectrum disorder (ASD) has been suggested to be associated with congenital factors, such as genomic mutations^1,2,3,4^ and prenatal, perinatal, and neonatal risk factors.^5,6,7^ In addition, abnormalities in brain morphology and function have been observed in children with ASD since early childhood.^8,9^ In studies conducted in 2019 and 2020, it has been reported that as a postnatal environmental factor, duration of screen time may be associated with ASD characteristics^10,11^ and brain morphology specific to ASD.^12^ Thus, screen time during infancy, a period of rapid development, may be one of the acquired factors that may be associated with ASD.

In 2019, the World Health Organization published guidelines on healthy physical activity, sedentary behavior, and sleep in children younger than 5 years of age, stating that children should not be exposed to screens at 1 year of age or younger.^13^ The American Academy of Pediatrics has also recommended that children should not be exposed to screens until they are 18 months of age; warnings about the adverse effects of screen exposure on the health of children have been issued.

In Japan, the most frequent age at diagnosis for ASD is 3.0 years.^14^ However, there are few large cohort studies that have focused on prolonged screen exposure and ASD in infancy. Furthermore, amid the recent outbreak of the COVID-19 pandemic, there has been a rapid change in lifestyles, with electronic devices being used as the main channels of communication and social interactions; thus, screen time among children has increased worldwide.^15,16,17^ Amid this social climate, examining the associations of screen exposure with a child’s health is an important public health issue.

Therefore, this study aimed to examine the association of screen exposure (an environmental factor) with the development of ASD during early childhood. To achieve this objective, we examined the association between screen time at 1 year of age and the presence or absence of ASD diagnosis at 3 years of age based on parental responses using data derived from a large Japanese birth cohort study (the Japan Environment and Children’s Study).

## Methods

### Study Design and Participants

We conducted a large birth cohort study in Japan. The Japan Environment and Children’s Study Group operated in collaboration with 15 regional centers across Japan. Its protocol was reviewed and approved by the Ministry of the Environment’s Institutional Review Board on Epidemiological Studies and the ethics committees of all the participating institutions. Approximately 100 000 pregnant women were recruited to participate in the study, and all of the participants provided written informed consent.^18^ The research was conducted in accordance with the Ethical Guidelines for Medical and Health Research Involving Human Subjects established by the Ministry of Education, Culture, Sports, Science and Technology and the Ministry of Health, Labour and Welfare. The recruitment period was from January 2011 to March 2014, and the data were analyzed during December 2020. The data used were derived from the strictly controlled jecs-ta-20190930-qsn data set, which was released in October 2019. The target population was selected as follows: first, the study included 100 304 live births of the 104 062 fetal records. Consequently, 382 stillbirths and 1254 miscarriages were excluded. We also excluded 2122 individuals with missing data once we calculated live births, stillbirths, and miscarriages. Next, we excluded 6449 children with cerebral palsy, a congenital condition, at 1 year of age, which may have influenced screen time. We also excluded 9825 children with missing data once we calculated congenital diseases or cerebral palsy. Finally, 84 030 mother-child dyads were included in the analysis (Figure).

**Figure.**
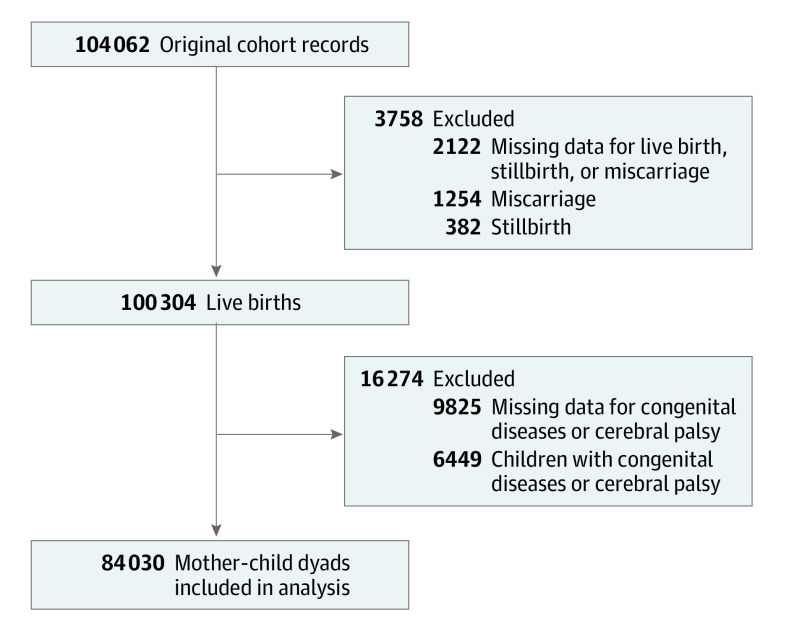
Selection Process for Participants

### Variables

The main exposure variable was screen time at 1 year of age, which was assessed using a questionnaire. When their child turned 1 year of age, the mothers were asked about the number of hours spent per day they let the child watch TV or DVDs. The responses were collected as variables and categorized as, “none (no screen time),” “less than 1 hour,” “1 hour or more but less than 2 hours,” “2 hours or more but less than 4 hours,” and “4 hours or more.” When the participating child turned 3 years of age, we asked mothers the same question. The outcome variable, ASD at 3 years of age, was assessed using a questionnaire. Specifically, mothers were asked the following question when their child turned 3 years of age: “Have they ever been diagnosed with autism spectrum disorder (eg, autism, pervasive developmental disorder, Asperger’s syndrome) by a doctor from the age of 2 years until now?” An option of 2 responses was provided, namely, with and without ASD, which were labeled as “yes (ASD)” or “no (no ASD),” respectively.

Previous studies have indicated that maternal nurturing attitudes and abuse may be associated with screen time.^19,20,21^ Therefore, the following factors served as adjustment variables: scores on the Kessler Psychological Distress Scale (K6) and Bonding Scale when the child was 1 year of age; depression, anxiety disorders, integration disorders, and other mental and neurological illnesses; mother’s age at delivery; and household income. In addition, when examining the association between ASD at 3 years of age and screen time at 1 year of age, we considered that predisposition to ASD may affect the outcome and may be attributable to reverse causality. We used participant scores on each of the 5 Ages and Stages Questionnaire [R] (ASQ-3) items, which served as adjustment variables to screen for ASD (communication, gross motor skills, fine motor skills, problem-solving, and personal-social scores) at 1 year of age. The ASQ-3 is valid and reliable in screening for developmental delays in children from 1 month of age to 5 1/2 of age, with age-appropriate questions. Having any of these 5 items is below the cutoff value, the line that prompts a visit to a specialist. Although this screening may not fully reflect the characteristics of ASD, its diagnostic accuracy is more than 80% and accurately detects ASD in most cases.^22,23^ Because ASD at 1 year of age is currently difficult to diagnose, the ASQ-3 was used in this study, and all of its 5 items were used as adjustment variables.

Each adjustment variable is described below. The Japanese version of the K6 scale was used to assess depressive tendencies,^24^ and the cutoff value was set at 5 points or more (Japanese version).^25^ A score above the cutoff value of K6 (≥5 points) indicated poor mental health. The Japanese version of the Bonding Scale was used to assess mother-child attachment. The scale consists of 10 questions and can yield a maximum score of 30 points; higher scores indicate more negative feelings toward one’s baby.^26^ However, no clear cutoff value was assigned for this scale; therefore, it was treated as a continuous variable. As younger mothers are at risk of abuse, a cutoff value of 19 years or younger at the time of delivery was used. Because poverty is also a risk factor for abuse, the poverty line reported by the Ministry of Health, Labour and Welfare (annual household income ≤¥ 1.27 million [$11 060.41 USD) was used as the standard,^27^ and the lowest income item (<¥ 2 million [$17 417.96 USD]) was used as the cutoff value for responses related to annual household income. The calculation of the poverty line reported by the Ministry of Health, Labour and Welfare is based on the standards of the Organization for Economic Cooperation and Development standards. For the 5 ASQ-3 items, we used the cutoff values specified for the Japanese version.^28^Additionally, because there are sex differences in ASD,^1,29^ sex was used as a stratification variable to examine sex differences in the results.

### Statistical Analysis

First, we aggregated the key variables by screen time at 1 year of age and at 3 years of age, and examined their attributes. Thereafter, odds ratios (ORs) and 95% CIs were calculated to examine the association between screen time and ASD. In addition, because sex differences have been reported in the prevalence of ASD, we examined the interaction by sex and then conducted a sex-stratified analysis. In the trend test in the adjusted model, the categorical variable (screen time) was statistically examined as an ordinal variable (continuous variable). Jonckheere-Terpstra tests were conducted to examine the association between screen time at 1 year of age and 3 years of age. The statistical significance level was set at .05 and 2-tailed. SPSS, version 27 (IBM), was used for statistical analysis.

## Results

### Aggregate Proportions of ASD at 3 Years of Age to Screen Time at 1 Year

A total of 84 030 mother-child dyads were analyzed. In 330 (0.4%) of the 84 030 children included in the analysis, ASD had been diagnosed at 3 years of age (Table 1). Of these, 251 were boys (76.0%) and 79 were girls (24.0%) (eTable 4 in Supplement 1). There were 83 237 responses for screen time at 1 year of age and 74 554 responses for screen time at 3 years of age (Table 1). Irrespective of whether the child had ASD at 3 years of age, at the age 1 year, less than 1 hour was the most commonly provided response for daily screen time (Table 1). The proportion of children with ASD increased as screen time increased (Table 1). The attributes of children and mothers by screen time are shown in Table 1 and eTable 3 in Supplement 1.

**Table 1.** Characteristics of the Mother-Child Dyads

Variables	No.	ASD at 3 y of age, No. (%)	Child's sex, No. (%)		Scores on the 5 items of the ASQ-3 at 1 y of age, No. (%)				
					Communication score <4.53	Gross motor score <9.43	Fine motor score <25.47	Problem solving score <15.37	Personal-social score <4.95
		ASD	Girls	Boys					
Screen time at 1 y of age, h									
No screen time	8541	19 (5.8)	4014 (9.9)	4527 (10.6)	14 (20.3)	535 (13.5)	520 (12.5)	434 (11.7)	134 (15.9)
<1	27 707	73 (22.3)	13 350 (32.9)	14 356 (33.7)	22 (31.9)	1339 (33.9)	1284 (30.8)	1026 (27.7)	254 (30.1)
1-<2	25 027	99 (30.2)	12 408 (30.5)	12 619 (29.6)	9 (13.0)	1147 (29.0)	1153 (27.6)	1089 (29.4)	248 (29.3)
2-<4	16 560	104 (31.7)	8207 (20.2)	8353 (19.6)	15 (21.7)	718 (18.2)	870 (20.8)	824 (22.3)	151 (17.9)
≥4	5402	33 (10.1)	2644 (6.5)	2757 (6.5)	9 (13.0)	211 (5.3)	348 (8.3)	329 (8.9)	58 (6.9)
Total	83 237	328	40 623	42 612	69	3950	4175	3702	845
Screen time at 3 y of age, h									
No screen time	1253	4 (1.2)	613 (1.7)	640 (1.7)	4 (6.5)	76 (2.1)	72 (1.9)	52 (1.5)	16 (2.1)
<1	17 874	58 (17.6)	8957 (24.6)	8917 (23.4)	13 (21.0)	971 (26.6)	839 (22.0)	702 (20.4)	196 (25.1)
1-<2	33 218	131 (39.7)	16 134 (44.3)	17 084 (44.8)	21 (33.9)	1560 (42.7)	1570 (41.3)	1434 (41.8)	330 (42.3)
2-<4	18 829	109 (33.0)	9114 (25.0)	9715 (25.5)	20 (32.3)	886 (24.2)	1079 (28.4)	1023 (29.8)	201 (25.8)
≥4	3380	28 (8.5)	1620 (4.4)	1760 (4.6)	4 (6.5)	162 (4.4)	245 (6.4)	222 (6.5)	37 (4.7)
Total	74 554	330	36 438	38 116	62	3655	3805	3433	780

Abbreviations: ASD, autism spectrum disorder; ASQ-3, Ages and Stages Questionnaire.

### Association Between Screen Time at 1 Year and ASD at 3 Years of Age

Logistic regression analysis of the association between screen time at 1 year and ASD at 3 years of age is shown in Table 2. Longer screen time at 1 year of age was associated with the statistically significantly higher odds of ASD at 3 years of age. Furthermore, the longer screen time at 1 year of age was associated with the statistically significantly higher odds of ASD at 3 years of age in boys. The distribution of screen time was similar across the sexes (Table 1). However, among girls, no association between screen time and ASD was found.

**Table 2.** Association Between Screen Time at 3 Years of Age and ASD, Stratified by Child's Sex

Variables	ASD at 3 y of age, ORs (95% CI)								
	Total			Boys			Girls		
	No.	Model 1, crude	Model 2, adjusted	No.	Model 1, crude	Model 2, adjusted	No.	Model 1, Crude	Model 2, adjusted
Screen time at 1 y of age,^a^ h	NA	(n = 74 179)	(n = 61 046)	NA	(n = 37 927)	(n = 31 034)	NA	(n = 36 252)	(n = 30 012)
No screen time	8541	1 [Reference]	1 [Reference]	4527	1 [Reference]	1 [Reference]	4014	1 [Reference]	1 [Reference]
<1	27 707	1.19 (0.72-1.97)	1.16 (0.66-2.03)	14 356	1.45 (0.79-2.64)	1.38 (0.71-2.69)	13 350	0.70 (0.27-1.82)	0.76 (0.27-2.18)
1-<2	25 027	1.81 (1.11-2.96)	1.81 (1.05-3.10)	12 619	2.08 (1.15-3.76)	2.16 (1.13-4.14)	12 408	1.37 (0.56-3.33)	1.28 (0.48-3.46)
2-<4	16 560	2.89 (1.77-4.71)	2.87 (1.68-4.91)	8353	3.40 (1.89-6.11)	3.48 (1.83-6.65)	8207	2.07 (0.85-5.04)	1.93 (0.72-5.20)
≥4	5402	2.80 (1.59-4.93)	2.64 (1.42-4.91)	2757	3.24 (1.66-6.35)	3.02 (1.44-6.34)	2644	2.05 (0.71-5.90)	2.15 (0.67-6.89)
* P* value for trend	NA	NA	<.001	NA	NA	<.001	NA	NA	.01
Screen time at 3 y of age^a^, h	NA	(n = 74 554)	(n = 61307)	NA	(n = 38116)	(n = 31148)	NA	(n = 36 438)	(n = 30 159)
No screen time	1253	1 [Reference]	1 [Reference]	640	1 [Reference]	1 [Reference]	613	1 [Reference]	1 [Reference]
<1	17 874	1.02 (0.37-2.81)	1.22 (0.38-3.93)	8917	1.03 (0.32-3.33)	1.30 (0.31-5.41)	8957	1.03 (0.14-7.79)	1.10 (0.14-8.53)
1-<2	33 218	1.24 (0.46-3.35)	1.38 (0.44-4.38)	17 084	1.29 (0.41-4.07)	1.60 (0.39-6.54)	16 134	1.06 (0.15-7.83)	0.96 (0.13-7.24)
2-<4	18 829	1.82 (0.67-4.94)	1.79 (0.56-5.70)	9715	1.85 (0.58-5.88)	2.04 (0.50-8.36)	9114	1.68 (0.23-12.44)	1.28 (0.17-9.78)
≥4	3380	2.61 (0.91-7.45)	2.40 (0.71-8.09)	1760	2.19 (0.64-7.47)	2.31 (0.52-10.19)	1620	3.80 (0.49-29.76)	2.64 (0.32-21.99)
* P* value for trend	NA	NA	.001	NA	NA	.005	NA	NA	.12
Interaction test by sex	<.001	NA	NA	NA	NA	NA	NA	NA	NA

Abbreviations: ASD, autism spectrum disorder; ASQ-3, Ages and Stages Questionnaire; NA, not applicable.

^a^ Adjustment for mothers’ scores on the Bonding Scale and Kessler Psychological Distress Scale when the child was 1 year of age, depression, anxiety disorders, integration disorders, other mental and neurological illnesses, mother’s age at delivery, household income, and scores on the 5 items of the ASQ-3 at 1 year of age.

### Association Between Screen Time at 3 Years and ASD at 3 Years of Age

The results of the trend test between screen time at 1 year of age and at 3 years of age showed that screen time at 1 year of age was statistically significantly associated with screen time at 3 years of age (Table 3). Namely, as the screen time increased, the proportion of children with ASD at 3 years of age also increased (Table 2). Moreover, logistic regression analysis reported that screen time at 3 years of age was not associated with ASD at 3 years of age (Table 3).

**Table 3.** Association Between Screen Time at 1 Year of Age and Screen Time at 3 Years of Age

Variable	Screen time at 3 y of age, No.						*P* value for trend^a^
	No screen	<1 h	1-<2 h	2-<4 h	≥4 h	Total	
Screen time at 1 y of age, h							
No screen time	595	3215	2846	916	92	7664	< .001
<1	385	8475	11 789	3835	305	24 789	NA
1-<2	164	4163	11 237	5949	588	22 101	NA
2-<4	75	1558	5792	6019	1130	14 574	NA
≥4	22	312	1281	1923	1227	4765	NA
Total	1241	17 723	32 945	18 642	3342	73 893	NA

Abbreviation: NA, not applicable.

^a^ Jonckheere-Terpstra test.

## Discussion

The main finding of this study was that, among boys, a statistically significant association was found between longer screen time at 1 year of age and ASD at 3 years of age, irrespective of potential maternal maltreatment or predisposition to ASD at 1 year of age. In this study, the prevalence of ASD among 3-year-old children was 0.4%, which is slightly lower than the prevalence of ASD among children younger than 5 years in Asia (0.70%).^30,31^ However, given that the prevalence of ASD increases with age, this study’s finding is comparable with those of previous studies. In addition, the sex ratio of children with ASD in this study is consistent with what has been observed in previous studies conducted in Japan and abroad.^14,32,33^ In Japan, parents who are concerned about their child having a developmental disability often visit medical institutions directly to receive a diagnosis. They could also receive a diagnosis after being advised to visit a medical institution for a possible developmental disability by an infant health checkup (conducted at 4 months, 1 ½ years, and 3 years of age), nursery school, kindergarten, or elementary school. Medical institutions make a diagnosis of ASD based on the *Diagnostic and Statistical Manual of Mental Disorders *(Fifth Edition).

Despite the World Health Organization and the American Academy of Pediatrics recommendations,^13^ 90% of the children in this study had been exposed to screens at 1 year of age. Few studies on screen time at 1 year of age have been reported in other countries. According to a survey conducted by the Cabinet Office in Japan, 85.7% of children younger than 1 year and 75.7% of 1-year-old children were using mobile phones, and many of them shared mobile phones with their parents.^34^ Thus, at 1 year of age, child-rearing environments may be associated with the development of ASD.

Multivariable analysis of the association between ASD and screen time was conducted to account for the influence of maternal maltreatment and children’s predisposition on the result. Among boys, irrespective of their predisposition to ASD at 1 year of age and maternal maltreatment factors, a longer screen time at 1 year of age was associated with ASD at 3 years of age.

In this study, we used the ASQ-3, which has a reported ASD diagnostic accuracy of more than 80%, to adjust for the predisposition of ASD at 1 year of age. However, the diagnostic criteria for ASD include, “Hyper- or hypo-reactivity to sensory input or unusual interest in sensory aspects of the environment” and a strong response to visual information such as lights or movement.^35^ This screening is limited because it does not fully reflect the characteristics of ASD. Therefore, we cannot deny the possibility of reverse causality. However, even in that case, screen time can be an effective indicator of ASD during early screening. In addition, this study examined the association between screen time at 1 year of age and ASD by excluding children who received a red flag on any of the 5 ASQ-3 items at 1 year of age (eTable 1 in Supplement 1), thereby helping the argument that screen time is a risk factor for the development of ASD.

The results of previous studies that have investigated the association between screen time and ASD in cross-sectional studies are not consistent.^32,36,37,38^ In this study, we examined the association between ASD at 3 years of age, at a single point in time, and screen time at the same age. The results showed no association between screen time and ASD at 3 years of age. This may have been due to the small size of the reference group; therefore, we set the reference group as less than 1 hour and conducted an additional analysis (eTable 2 in Supplement 1). Consequently, we found that there is a statistically significant difference between boys reported to have 2 to less than 4 hours of screen time. However, the results of the estimates were close to the reference values before they were changed. This indicates that the association of screen time at 1 year of age with ASD diagnosis is still greater than at 3 years of age. This may be because the association with environmental factors on brain development varies with age.

In addition to genetic factors, the role of environmental factors has been noted in ASD. Electromagnetic fields have been cited as an environmental factor associated with health and screen exposure.^39^ Experiments using mice have demonstrated that exposure to high-frequency electromagnetic fields affects neurotransmitters^40^ and behavior (hyperactivity and memory impairment)^41^ in mice during the developmental period. Additionally, several molecular networks as genetic factors have been associated with the development of ASD, and the core of these molecular networks include α-amino-3-hydroxy-5-methyl-4-isoxazolepropionic acid receptor (AMPA receptor), protein kinase B (AKT), repressor activator protein 1 (RAP1), γ-aminobutyric acid (GABA), extracellular signal-regulated kinases 1/2 (ERK1/2), methyl-CpG-binding protein 2 (MECP2), brain-derived neurotrophic factor (BDNF), activator protein 1 (AP-1), phosphatase and tensin homolog (PTEN), and ras protein/mitogen-activated protein kinase (RAS/MAPK). Previous studies have reported that low-frequency and high-frequency electrical stimulation, microwave irradiation, and light stimulation of AMPA receptors,^42^ Rfn2,^43^ GABA,^44^ MECP2,^45^ and BDNF^1,46,47,48^ are associated with autismlike symptoms. In particular, in infancy when neurodevelopment is active, environmental factors such as electrical stimulation through screens and light stimulation from vision may affect neurodevelopment and de novo sequence alterations.

In this study, we examined the interaction by sex and then conducted a sex-differentiated analysis. The results suggested an association between screen time and ASD only in boys, even if boys and girls have similar screen times (eTable 4 in Supplement 1). This result could be due to the higher prevalence of the disorder in boys. Previous studies on the genetic factors that contribute to the development of ASD have not yet explained the male predominance in ASD.^1^ However, among the factors listed as the core genes responsible for the development of ASD, sex differences have been reported for brain-derived neurotrophic factor^49,50^ and MECP2.^51^ Thus, genetic factors may be involved in the observed sex differences in the association between ASD and screen time.

### Strengths and Limitations

The strength of this study is that it provides new insights into the association of screen time with the onset of ASD in early childhood, which had not yet been examined. Furthermore, the results of this study are reliable because the analysis was conducted using a large birth cohort data set representative of the Japanese population.^52^

 A limitation of this study is that ASD (outcome) and screen time (exposure) were assessed based on parental reports, which may have led to reporting bias. Data on the exact amount of time children are gazing at the screen are not available, and it may differ from what parents report. Medical institutions diagnose ASD based on the *Diagnostic and Statistical Manual of Mental Disorders* (Fifth Edition) but only when ASD can be diagnosed with certainty because it is still in a developmental stage when children are 3 years of age. Therefore, mild cases may not be diagnosed as ASD at 3 years of age, when the child is at a developmental stage, and may be observed in subsequent years. As a result, the study may be biased toward children with severe ASD. To account for potential risks, we conducted a multivariable analysis with ASD predisposition (ASQ-3) at 1 year of age. Additionally, external (eg, prenatal, living, and childcare environment) and internal factors (eg, genetic factors, diseases, and disabilities) other than screen time, which were examined as exposure factors in this study, were not adequately considered. Therefore, this study conducted a sensitivity analysis of unmeasured confounding as an additional analysis (the E-value),^53,54,55,56,57^ and it was found that the effect of residual confounding remained (eTable 5 in Supplement 1). Further research is needed to examine other factors involved in the association between ASD and screen time, and the combination of factors at a given time of the year associated with increased risk of ASD.

## Conclusions

In this cohort study, even after accounting for predisposition to ASD at 1 year of age and maternal maltreatment factors, longer screen time at 1 year of age was associated with ASD at 3 years of age in boys. With the rapid increase in the use of devices, it is necessary to review its health effects on infants and control excessive screen time.

## Notes

Supplement 1. **eTable 1.** Association between ASD at 3 years of age and screen time, stratified by child’s sex (excluding those who had at least one red flag on any of the five ASQ items) **eTable 2.** Association between ASD at 3 years of age and screen time at 3, stratified by child’s sex **eTable 3.** Characteristics of the mother–child dyads **eTable 4.** Cross-tabulation and results of the Jonckheere-Terpstra Test **eTable 5.** Sensitivity analysis of unmeasured confounding

Supplement 2. Nonauthor collaborators
