# Supplementary material for: Association Between Screen Time Exposure in Children at 1 Year of Age and Autism Spectrum Disorder at 3 Years of Age: The Japan Environment and Children’s Study
Source: JAMA Pediatr. 2022 Jan 31;176(4):384–91. doi: 10.1001/jamapediatrics.2021.5778 (PMC8804971; doi:10.1001/jamapediatrics.2021.5778)
Supplement: Supplement 1. — eTable 1. Association between ASD at 3 years of age and screen time, stratified by child’s sex (excluding those who had at least one red flag on any of the five ASQ items) eTable 2. Association between ASD at 3 years of age and screen time at 3, stratified by child’s sex eTable 3. Characteristics of the mother–child dyads eTable 4. Cross-tabulation and results of the Jonckheere-Terpstra Test eTable 5. Sensitivity analysis of unmeasured confounding [file jamapediatr-e215778-s001.pdf]

Supplemental Online Content

Kushima M, Kojima R, Shinohara R, et al; the Japan Environment and Children's Study Group. Association between screen time exposure in children at 1 year of age and autism spectrum disorder at 3 years of age: the Japan Environment and Children's Study. *JAMA Pediatr*. Published online January 31, 2022. doi:10.1001/jamapediatrics.2021.5778

eTable 1. Association between ASD at 3 years of age and screen time, stratified by child's sex (excluding those who had at least one red flag on any of the five ASQ items)
eTable 2. Association between ASD at 3 years of age and screen time at 3, stratified by child's sex
eTable 3. Characteristics of the mother–child dyads
eTable 4. Cross-tabulation and results of the Jonckheere-Terpstra Test
eTable 5. Sensitivity analysis of unmeasured confounding

This supplemental material has been provided by the authors to give readers additional information about their work.

© 2022 Kushima M et al. *JAMA Pediatrics.*

eTable 1. Association between ASD at 3 years of age and screen time, stratified by child’s sex (excluding those who had at least one red flag on any of the five ASQ items)

Variables	ASD at 3 years of age								
	Total			Boys			Girls		
		Model 1 (Crude)	Model 2 (Adjusted)		Model 1 (Crude)	Model 2 (Adjusted)		Model 1 (Crude)	Model 2 (Adjusted)
	n	ORs (95％CI)	ORs (95％CI)	n	ORs (95％CI)	ORs (95％CI)	n	ORs (95％CI)	ORs (95％CI)
Screen time at 1 years of age†		（n = 57245）	（n = 52710）		（n = 28779）	（n = 26472）		（n = 28466）	（n = 26238）
No screen time	6324	1.00 (reference)	1.00 (reference)	3267	1.00 (reference)	1.00 (reference)	3057	1.00 (reference)	1.00 (reference)
<1 h	21361	1.00 (0.51 to 1.96)	1.04 (0.51 to 2.09)	10947	1.29 (0.57 to 2.94)	1.38 (0.57 to 3.34)	10414	0.51 (0.15 to 1.74)	0.52 (0.15 to 1.77)
1－<2 h	19071	1.79 (0.94 to 3.42)	1.70 (0.87 to 3.35)	94339	2.20 (0.99 to 4.88)	2.34 (0.99 to 5.52)	9632	1.20 (0.40 to 3.61)	0.92 (0.30 to 2.88)
2－＜4 h	12498	2.85 (1.50 to 5.42)	2.78 (1.42 to 5.44)	6196	3.78 (1.71 to 8.35)	3.87 (1.65 to 9.09)	6302	1.47 (0.47 to 4.55)	1.39 (0.44 to 4.34)
≧4 h	3984	2.80 (1.33 to 5.89)	2.28 (1.03 to 5.06)	1979	3.42 (1.38 to 8.48)	2.69 (0.99 to 7.34)	2005	1.92 (0.52 to 7.16)	1.81 (0.48 to 6.86)
Screen time at 3 years of age†		（n = 57532）	（n = 52942）		（n = 28909）	（n = 26568）		（n = 28623）	（n = 26374）
<1 h‡	14774	1.00 (reference)	1.00 (reference)	7262	1.00 (reference)	1.00 (reference)	7512	1.00 (reference)	1.00 (reference)
1－<2 h	25830	1.42 (0.94 to 2.16)	1.33 (0.87 to 2.04)	13102	1.39 (0.86 to 2.23)	1.31 (0.80 to 2.13)	12728	1.43 (0.59 to 3.46)	1.32 (0.54 to 3.22)
2－＜4 h	14432	2.15 (1.40 to 3.30)	1.90 (1.23 to 2.96)	7287	2.13 (1.31 to 3.46)	1.96 (1.19 to 3.23)	7145	2.11 (0.85 to 5.22)	1.64 (0.64 to 4.20)

© 2022 Kushima M et al. *JAMA Pediatrics.*

≧4 h	2496	2.88 (1.55 to 5.33)	2.42 (1.27 to 4.59)	1258	2.42 (1.15 to 5.07)	1.98 (0.91 to 4.35)	1238	4.35 (1.38 to 13.72)	3.69 (1.16 to 11.80)

†Adjustment: Mothers' scores on the Bonding Scale and K6 scores when the child at 1 year of age, depression, anxiety disorders, integration disorders, other mental and neurological illnesses, mother's age at birth, and household income, scores on the 5 items of the ASQ-3 at 1 year of age.

‡ Since complete separation occurred, the reference value was set to less than one hour.

ORs = Odds ratios; 95% CI = 95% confidence intervals; ASD: Autism spectrum disorder; ASQ-3: Ages and Stages Questionnaire

© 2022 Kushima M et al. *JAMA Pediatrics.*

eTable 2. Association between ASD at 3 years of age and screen time at 3, stratified by child's sex									
Variables	ASD at 3 years of age								
	Total			Boys			Girls		
		Model 1 (Crude)	Model 2 (Adjusted)		Model 1 (Crude)	Model 2 (Adjusted)		Model 1 (Crude)	Model 2 (Adjusted)
	n	ORs (95％CI)	ORs (95％CI)	n	ORs (95％CI)	ORs (95％CI)	n	ORs (95％CI)	ORs (95％CI)
Screen time at 3 years of age†		（n = 74554）	（n = 61307）		（n = 38116）	（n = 31148）		（n = 36438）	（n = 30159）
<1 h	19127	1.00 (reference)	1.00 (reference)	9557	1.00 (reference)	1.00 (reference)	9570	1.00 (reference)	1.00 (reference)
1－<2 h	33218	1.22 (0.90 to 1.65)	1.15 (0.83 to 1.60)	17084	1.25 (0.89 to 1.78)	1.25 (0.86 to 1.83)	16134	1.04 (0.56 to 1.92)	0.88 (0.46 to 1.69)
2－＜4 h	18829	1.79 (1.31 to 2.45)	1.49 (1.06 to 2.10)	9715	1.80 (1.26 to 2.59)	1.60 (1.07 to 2.38)	9114	1.64 (0.88 to 3.08)	1.17 (0.59 to 2.33)
≧4 h	3380	2.57 (1.64 to 4.02)	2.00 (1.21 to 3.30)	1760	2.14 (1.24 to 3.69)	1.81 (0.98 to 3.32)	1620	3.71 (1.68 to 8.19)	2.42 (0.97 to 6.01)

†Adjustment: Mothers' scores on the Bonding Scale and K6 scores when the child at 1 year of age, depression, anxiety disorders, integration disorders, other mental and neurological illnesses, mother's age at birth, and household income, scores on the 5 items of the ASQ-3 at 1 year of age.
ORs = Odds ratios; 95% CI = 95% confidence intervals; ASD: Autism spectrum disorder; ASQ-3: Ages and Stages Questionnaire

© 2022 Kushima M et al. *JAMA Pediatrics.*

eTable 3. Characteristics of the mother–child dyads										
Variables			Mother's Bonding score when her child was 1 year of age	Mother's K6 scores when her child was 1 year of age	Mother's history of mental illness				Mother's age at birth	Household income
					Depression	Anxiety disorders	Integration disorders	Other mental neurological illnesses		
			Average/ Median / Max/ Standard deviation	≧ 5 points	Yes	Yes	Yes	Yes	＜ 20 y	＜ 2 million yen
			n (%)	n (%)	n (%)	n (%)	n (%)	n (%)	n (%)	n (%)
Screen time at 1 year of age	No screen time	（n = 8541）	1.6/ 1.0/ 0.0/ 2.2	1654 (9.9)	230 (9.5)	247 (11.1)	10 (7.6)	90 (11.1)	33 (5.7)	447 (11.0)
	< 1 h	（n = 27707）	1.7/ 1.0/ 0.0/ 2.1	5473 (29.9)	699 (28.9)	630 (28.1)	40 (30.3)	239 (29.6)	165 (28.6)	1284 (31.6)
	1－ < 2 h	（n = 25027）	2.0/ 1.0/ 0.0/ 2.2	5525 (30.2)	720 (29.8)	689 (30.7)	35 (26.5)	223 (27.6)	178 (30.9)	1115 (27.4)

© 2022 Kushima M et al. *JAMA Pediatrics.*

	2－ < 4 h	（n = 16560）	2.2/ 2.0/ 0.0/ 2.5	4143 (22.6)	566 (23.4)	516 (23.0)	37 (28.0)	183 (22.6)	129 (22.4)	871 (21.4)
	≧ 4 h	（n = 5402）	2.3/ 2.0/ 0.0/ 2.5	1526 (8.3)	200 (8.3)	163 (7.3)	10 (7.6)	73 (9.0)	71 (12.3)	350 (8.6)
	Total	（n = 83237）	2.5/ 2.0/ 0.0/ 2.7	18321	2415	2245	132	808	576	4067
Screen time at 3 year of age	No screen time	（n = 1253）	2.0/ 1.0/ 0.0/ 2.4	1654 (9.9)	59 (2.8)	36 (1.9)	2 (1.9)	14 (2.0)	9 (2.2)	72 (2.1)
	< 1 h	（n = 17874）	1.8/ 1.0/ 0.0/ 2.4	5473 (29.9)	447 (21.3)	427 (22.2)	20 (18.5)	159 (22.9)	89 (21.3)	809 (23.6)
	1－ < 2 h	（n = 33218）	1.7/ 1.0/ 0.0/ 2.2	5525 (30.2)	873 (41.7)	807 (42.0)	49 (45.4)	273 (39.3)	191 (45.7)	1485 (43.2)
	2－ < 4 h	（n = 18829）	1.9/ 1.0/ 0.0/ 2.2	4143 (22.6)	571 (27.3)	545 (28.3)	28 (25.9)	205 (29.5)	88 (21.1)	865 (25.2)
	≧ 4 h	（n = 3380）	2.2/ 1.0/ 0.0/ 2.4	1526 (8.3)	144 (6.9)	108 (5.6)	9 (8.3)	43 (6.2)	41 (9.8)	203 (5.9)
	Total	（n = 74554）	2.5/ 2.0/ 0.0/ 2.7	18321	2094	1923	108	694	418	3434

© 2022 Kushima M et al. *JAMA Pediatrics.*

eTable 4. Cross-tabulation and results of Jonckheere-Terpstra Test																			
Variables			Screen time at 1 year of age							Screen time at 3 years of age							Child's sex		
			No screen time	< 1 h	1—< 2 h	2—< 4 h	≧ 4 h	Total	P for trend test†	No screen time	< 1 h	1—< 2 h	2—< 4 h	≧ 4 h	Total	P for trend test†	Girl	Boy	Total
Total	ASD	n	19	73	99	104	33	328	< 0.001	4	58	131	109	28	330	< 0.001	79	251	330
		(%)	(5.8)	(22.3)	(30.2)	(31.7)	(10.1)			(1.2)	(17.6)	(39.7)	(33.0)	(8.5)			(24.0)	(76.0)	
	Non-ASD	n	7669	24785	22099	14538	4760	73851		1249	17816	33087	18720	3352	74224		36498	38018	74516
		(%)	(10.4)	(33.6)	(29.9)	(19.7)	(6.4)			(1.7)	(24.0)	(44.6)	(25.2)	(4.5)			(49.0)	(51.0)	
	Total	n	7688	24858	22198	14642	4793	74179		1253	17874	33218	18829	3380	74554		36577	38269	74846
		(%)	(10.4)	(33.5)	(29.9)	(19.7)	(6.4)			(1.7)	(24.0)	(44.6)	(25.3)	(4.5)			(49.0)	(51.0)	
Boy	ASD	n	13	59	74	79	25	250	< 0.001	3	43	103	84	18	251	< 0.001			
		(%)	(5.2)	(23.6)	(29.6)	(31.6)	(10.0)			(1.2)	(17.1)	(41.0)	(33.5)	(7.2)					
	Non-ASD	n	4070	12786	11126	7282	2413	37677		637	8874	16981	9631	1742	37865				

© 2022 Kushima M et al. *JAMA Pediatrics.*

		(%)	(10.8)	(33.9)	(29.5)	(19.3)	(6.4)			(1.7)	(23.4)	(44.8)	(25.4)	(4.6)					
	Total	n	4083	12845	11200	7361	2438	37927		640	8917	17084	9715	1760	38116				
		(%)	(10.8)	(33.9)	(29.5)	(19.4)	(6.4)			(1.7)	(23.4)	(44.8)	(25.5)	(4.6)					
Girl	ASD	n	6	14	25	25	8	78	< 0.001	1	15	28	25	10	79	< 0.001			
		(%)	(7.7)	(17.9)	(32.1)	(32.1)	(10.3)			(1.3)	(19.0)	(35.4)	(31.6)	(12.7)					
	Non-ASD	n	3599	11999	10973	7256	2347	36174		612	8942	16106	9089	1610	36359				
		(%)	(9.9)	(33.2)	(30.3)	(20.1)	(6.5)			(1.7)	(24.6)	(44.3)	(25.0)	(4.4)					
	Total	n	3605	12013	10998	7281	2355	36252		613	8957	16134	9114	1620	36438				
		(%)	(9.9)	(33.1)	(30.3)	(20.1)	(6.5)			(1.7)	(24.6)	(44.3)	(25.0)	(4.4)					

†Jonckheere Terpstra test

© 2022 Kushima M et al. *JAMA Pediatrics.*

eTable 5. Sensitivity analysis of unmeasured confounding									
Variables	Total			Boys			Girls		
	E-value for point estimate	E-value for confidence interval		E-value for point estimate	E-value for confidence interval		E-value for point estimate	E-value for confidence interval	
		Lower limit	Upper limit		Lower limit	Upper limit		Lower limit	Upper limit
Screen time at 1 year of age†									
No screen time									
< 1 h	1.59	11.98	3.48	2.10	2.17	4.82	1.96	6.87	3.78
1－ < 2 h	3.02	1.28	5.65	3.74	1.51	7.75	1.88	3.59	6.38
2－ < 4 h	5.19	2.75	9.29	6.42	3.06	12.78	3.27	2.12	9.87
≧ 4 h	4.72	2.19	9.29	5.49	2.24	12.16	3.72	2.35	13.26
Screen time at 3 years of age†									
No screen time									
< 1 h	1.74	4.70	7.32	1.92	5.91	10.29	1.43	13.77	16.54
1－ < 2 h	2.10	3.97	8.23	2.58	4.57	12.56	1.25	14.87	13.96
2－ < 4 h	2.98	2.97	10.88	3.50	2.84	16.20	1.88	11.24	19.05
≧ 4 h	4.23	2.17	15.66	4.05	3.26	19.87	4.72	5.70	43.47
†Adjustment: Mothers' scores on the Bonding Scale and K6 scores when the child at 1 year of age, depression, anxiety disorders, integration disorders, other mental and neurological illnesses, mother's age at birth, and household income, scores on the 5 items of the ASQ-3 at 1 year of age.									

© 2022 Kushima M et al. *JAMA Pediatrics.*

E-value = OR+√ OR (OR-1)									

© 2022 Kushima M et al. *JAMA Pediatrics.*
