# Supplementary material for: Association Between Screen Time Exposure in Children at 1 Year of Age and Autism Spectrum Disorder at 3 Years of Age: The Japan Environment and Children’s Study
Source: JAMA Pediatr. 2022 Jan 31;176(4):384–91. doi: 10.1001/jamapediatrics.2021.5778 (PMC8804971; doi:10.1001/jamapediatrics.2021.5778)
Supplement: Supplement 2. — Nonauthor collaborators [file jamapediatr-e215778-s002.pdf]

Supplemental Online Content: Nonauthor Collaborators

*Indicates required information. Only first name, last name, and suffix will appear in PubMed.

*Group Name(s): the Japan Environment and Children's Study (JECS) Group							
***First Name and Middle Initial(s)**	***Last Name**	***Suffix (eg, Jr, III)**	Academic Degrees	Institution	Location (city, state/province, country)	Role or Contribution, eg, chair, principal investigator	Group (if more than 1 Group listed in the byline) and/or Subgroup (eg, Steering Committee)
Michihiro	Kamijima		MD PhD	Nagoya City University	Nagoya, Aichi, Japan	principal investigator	
Shin	Yamazaki		PhD	National Institute for Environmental Studies	Tsukuba, Ibaraki, Japan	Project design, data collection and review	
Yukihiro	Ohya		MD PhD	National Center for Child Health and Development	Setagaya, Tokyo, Japan	Project design, data collection and review	
Reiko	Kishi		MD PhD	Hokkaido University	Sapporo, Hokkaido, Japan	Project design, data collection and review	
Nobuo	Yaegashi		MD PhD	Tohoku University	Sendai, Miyagi, Japan	Project design, data collection and review	
Koichi	Hashimoto		MD PhD	Fukushima Medical University	Fukushima, Japan	Project design, data collection and review	
Chisato	Mori		MD PhD	Chiba University	Chiba, Japan	Project design, data collection and review	
Shuichi	Ito		MD PhD	Yokohama City University	Yokohama, Kanagawa, Japan	Project design, data collection and review	
Zentaro	Yamagata		MD PhD	University of Yamanashi	Chuo, Yamanashi, Japan	Project design, data collection and review	
Hidekuni	Inadera		MD PhD	University of Toyama	Toyama, Japan	Project design, data collection and review	

Supplemental Online Content: Nonauthor Collaborators

*Indicates required information. Only first name, last name, and suffix will appear in PubMed.

***First Name and Middle Initial(s)**	***Last Name**	***Suffix (eg, Jr, III)**	Academic Degrees	Institution	Location (city, state/province, country)	Role or Contribution, eg, chair, principal investigator	Group (if more than 1 Group listed in the byline) and/or Subgroup (eg, Steering Committee)
Takeo	Nakayama		MD PhD	Kyoto University	Kyoto, Japan	Project design, data collection and review	
Hiroyasu	Iso		MD PhD	Osaka University	Suita, Osaka, Japan	Project design, data collection and review	
Masayuki	Shima		MD PhD	Hyogo College of Medicine	Nishinomiya, Hyogo, Japan	Project design, data collection and review	
Hiroshige	Nakamura		MD PhD	Tottori University	Yonago, Tottori, Japan	Project design, data collection and review	
Narufumi	Suganuma		MD PhD	Kochi University	Nankoku, Kochi, Japan	Project design, data collection and review	
Koichi	Kusuhara		MD PhD	University of Occupational and Environmental Health	Kitakyushu, Fukuoka, Japan	Project design, data collection and review	
Takahiko	Katoh		MD PhD	Kumamoto University	Kumamoto, Japan	Project design, data collection and review	
